# Supplementary material for: Enhanced pericyte-endothelial interactions through NO-boosted extracellular vesicles drive revascularization in a mouse model of ischemic injury
Source: Nat Commun. 2023 Nov 13;14:7334. doi: 10.1038/s41467-023-43153-x (PMC10643472; doi:10.1038/s41467-023-43153-x)
Supplement: Supplementary file 1 — Supplementary Information [file 41467_2023_43153_MOESM1_ESM.pdf]

Supplementary Information

Enhanced pericyte-endothelial interactions through NO-boosted extracellular vesicles drive revascularization in a mouse model of ischemic injury

Ling Guo[†*1,2], *Qiang Yang*[†1], *Runxiu Wei*[1], *Wenjun Zhang*[1], *Na Yin*[1], *Yuling Chen*[1], *Chao Xu*[2], *Changrui Li*[3], *Randy P. Carney*[*4], *Yuanpei Li*[*5], *Min Feng*[*1]

[1]School of Pharmaceutical Sciences, Sun Yat-sen University; University Town, Guangzhou, 510006, P.R. China.

[2] Key Laboratory of Tropical Biological Resources of Ministry of Education, School of Pharmaceutical Sciences, Hainan University; Haikou, 570228, P. R. China.

[3] Guangzhou Zhixin High School, Zhixin South Road, Guangzhou, 510080, P.R. China.

[4] Department of Biomedical Engineering, University of California Davis; Davis, CA, 95616, USA.

[5]Department of Biochemistry and Molecular Medicine, UC Davis Comprehensive Cancer Center, University of California Davis; Davis, CA, 95616, USA.

[†]These authors contributed equally to this work.

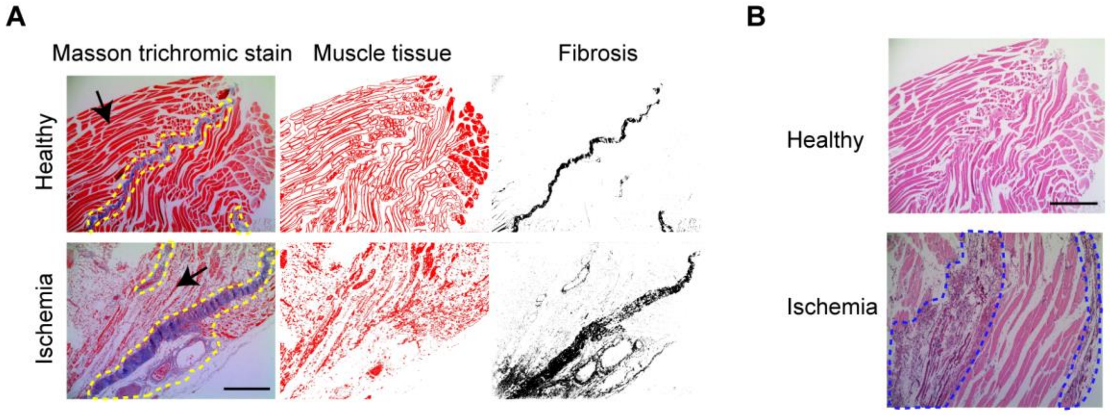

Supplementary Figure 1. Pathological images of ischemic hindlimb muscle. (A) Masson's trichrome staining of hindlimb sections. Muscle tissue was stained red. Light blue staining for collagen indicates fibrosis (indicated by dotted lines). Scale bar, 1000 μm. (B) Representative H&E-stained sections of hindlimb muscles showing inflammatory cell infiltration (indicated by dotted lines). Scale bar, 1000 μm.

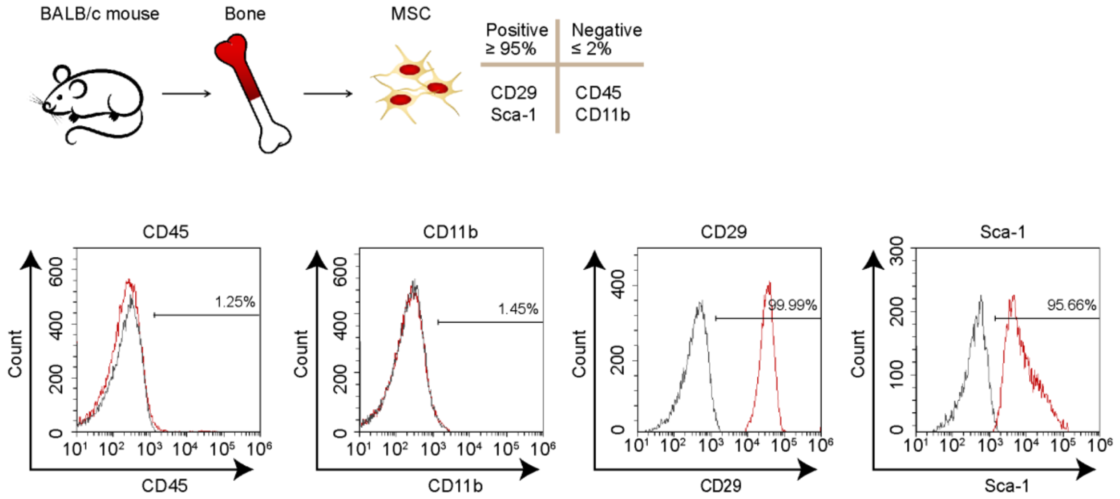

Supplementary Figure 2. Flow cytometry analysis of MSCs shown as percent of $CD29^{+}Sca\text{-}1^{+}CD45^{-}CD11b^{-}$ cells.

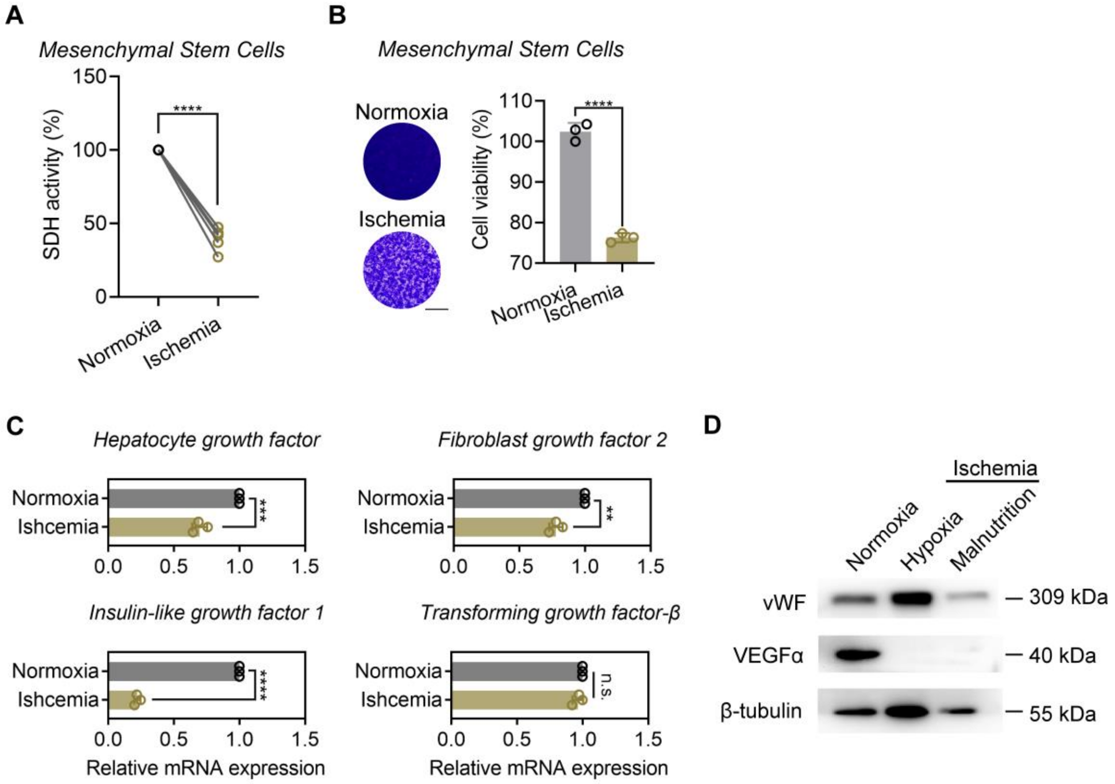

Supplementary Figure 3. Cellular activities and paracrine abilities of MSCs in the ischemic microenvironment. (A) Mitochondrial succinate dehydrogenase activities of MSCs were measured using an MTT assay at 6 h (n = 6). (B) Cell viability of MSCs exposed to ischemic conditions was determined by crystal violet staining at 24 h (n = 3). Scale bar, 1000 μm. (C) The expression of angiogenic growth factors (HGF, FGF2, IGF1 and TGF-β) was analyzed by qRT-PCR (n = 3). (D) Western blot of vWF, VEGFα and β-tubulin with protein lysates from MSCs. Data are mean ± SD, ** is $P < 0.01$, *** is $P < 0.001$, **** is $P < 0.0001$, n.s. is $P > 0.05$ by Student's t test.

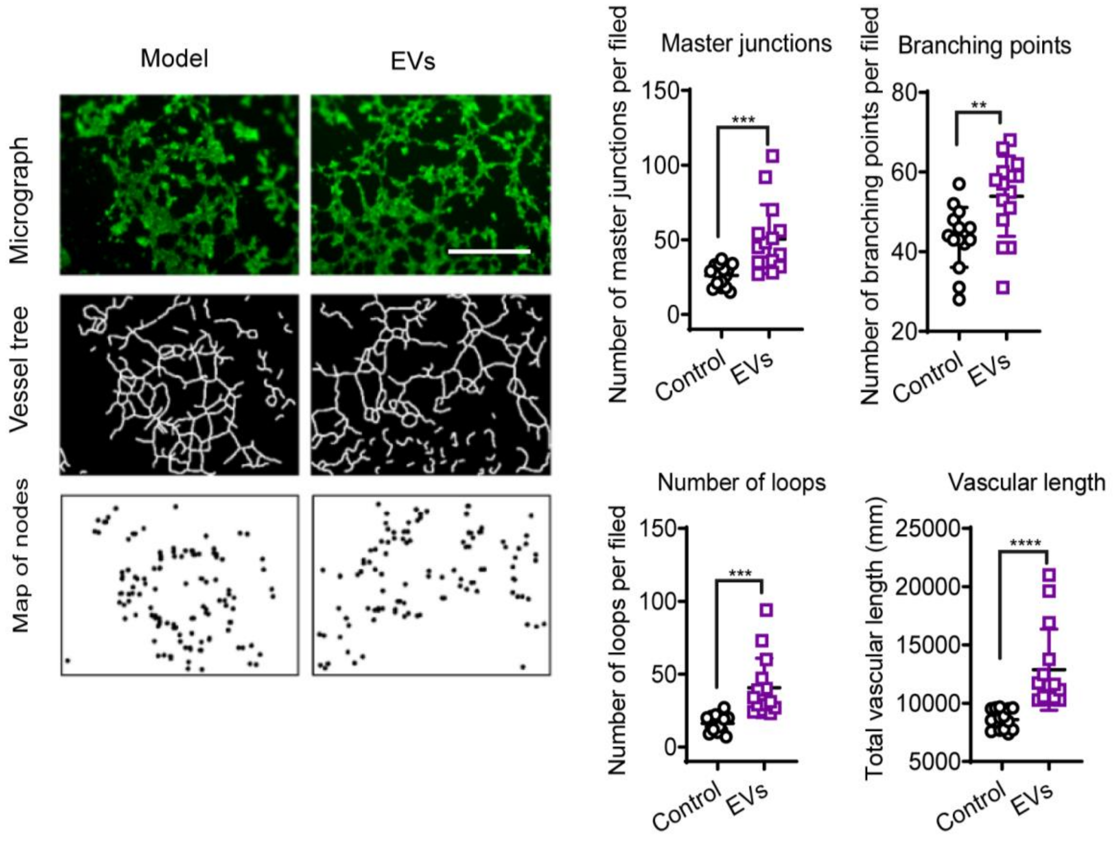

Supplementary Figure 4. Representative images of endothelial tube formation and quantification of vascular progression (number of loops, vascular length, number of branching points and number of master junctions) at 4 h after seeding (n = 15). Scale bar, 1000 μm. Data are mean ± SD, ** is $P < 0.01$, *** is $P < 0.001$, **** is $P < 0.0001$ by Student’s t-test.

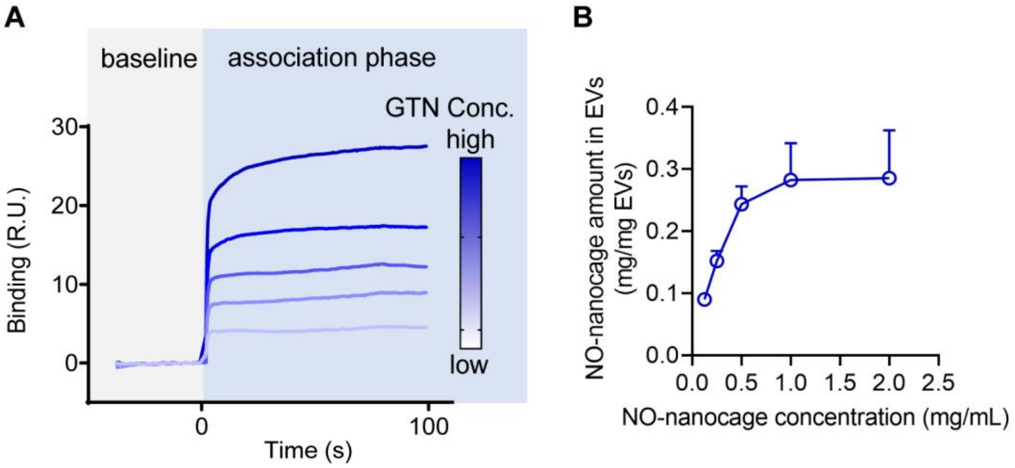

Supplementary Figure 5. Binding affinity of the components in n-BANKs. (A) Surface plasmon resonance (SPR) determination of GTN binding affinity to albumin. (B) Binding curve of NO-nanocages to EVs (n = 5). Data are mean ± SD.

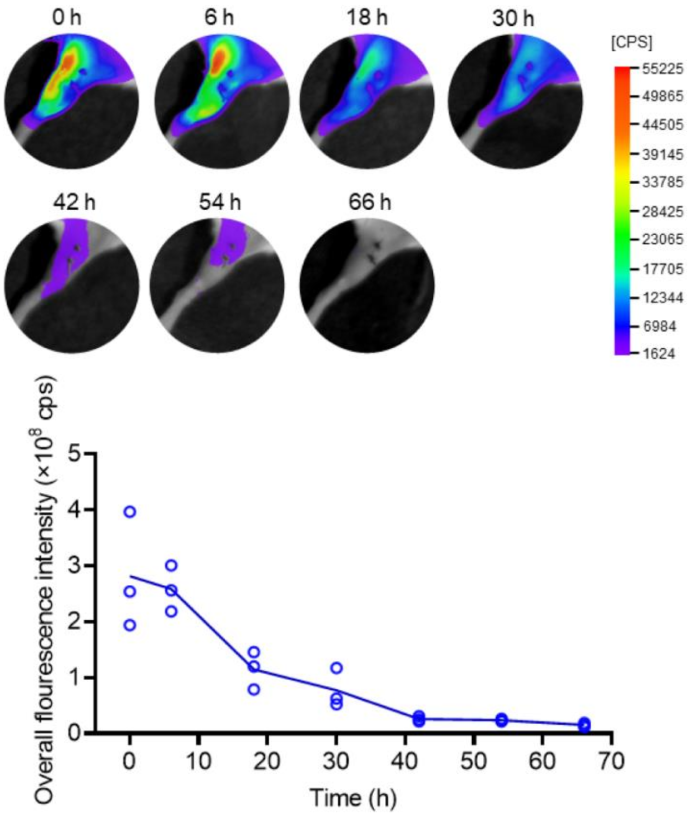

Supplementary Figure 6. Live imaging and quantitation of fluorescence intensity of FITC-labeled n-BANKs after i.m. injection at the indicated times (n = 3).

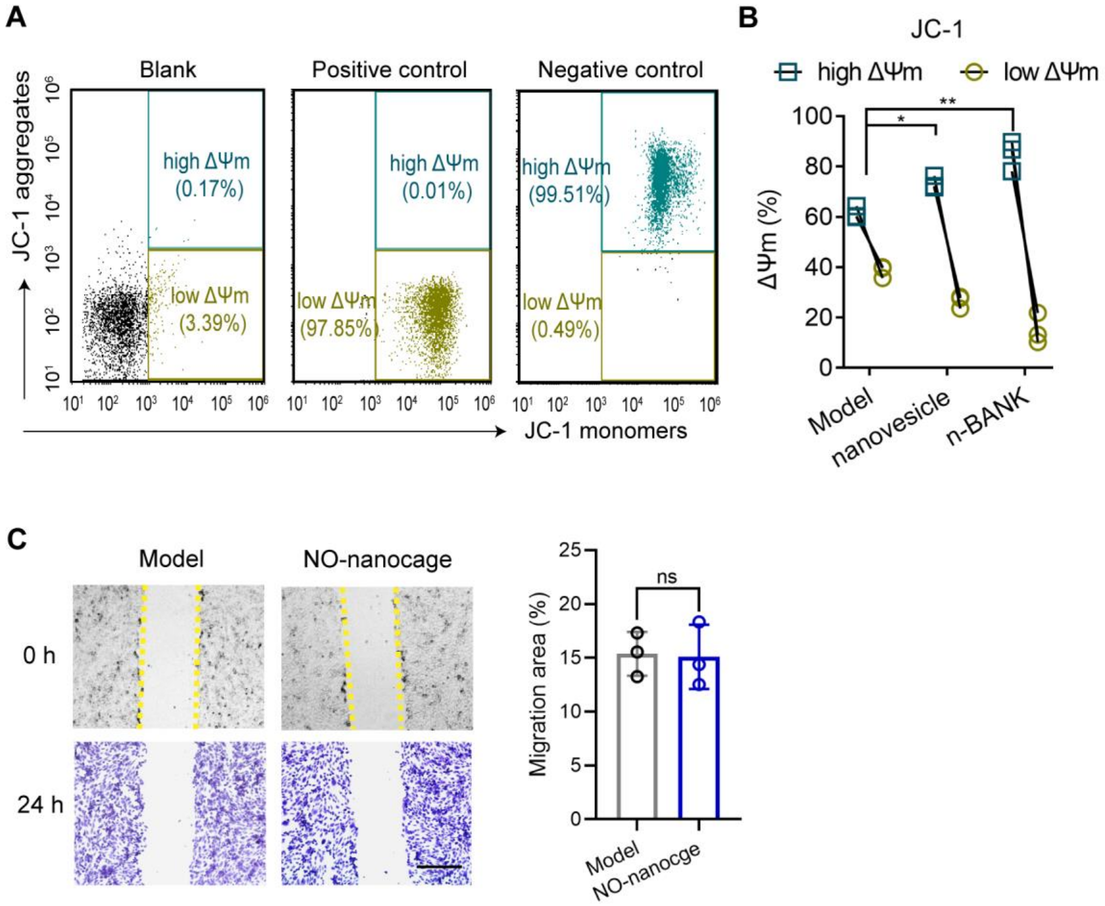

Supplementary Figure 7. (A) Flow cytometry analysis of SVEC4-10 cells. An example of the gating strategy for the analysis of JC-1-stained cells. (B) Quantification of mitochondrial membrane potential in SVEC4-10 cells. SVEC4-10 cells were exposed to hypoxia for 12 hours prior to treatment (n = 3). (C) Representative photomicrographs and quantification of a scratch-cell motility assay of SVEC4-10 cells under ischemic conditions for the indicated times (n = 3). Scale bar, 400 μm. Data are mean ± SD, * is $P < 0.05$, ** is $P < 0.01$ and n.s. is $P > 0.05$ by two-way ANOVA test or Student's t-test.

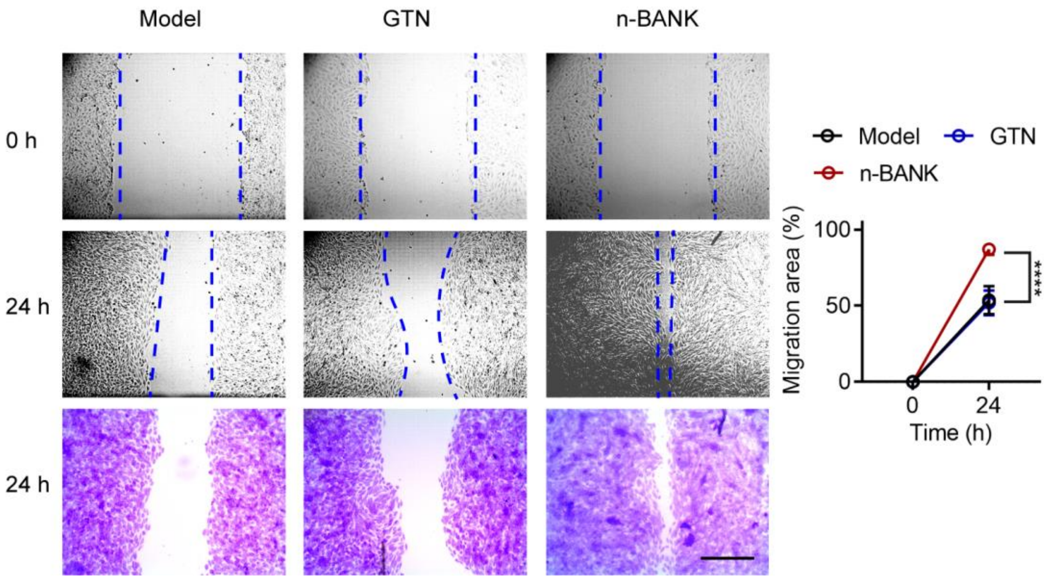

Supplementary Figure 8. Representative photomicrographs and quantification of a scratch-cell motility assay of SVEC4-10 cells under normoxia for 24 h (n = 6). Scale bar, 400 μm. Data are mean ± SD, **** is $P < 0.0001$ by one-way ANOVA test.

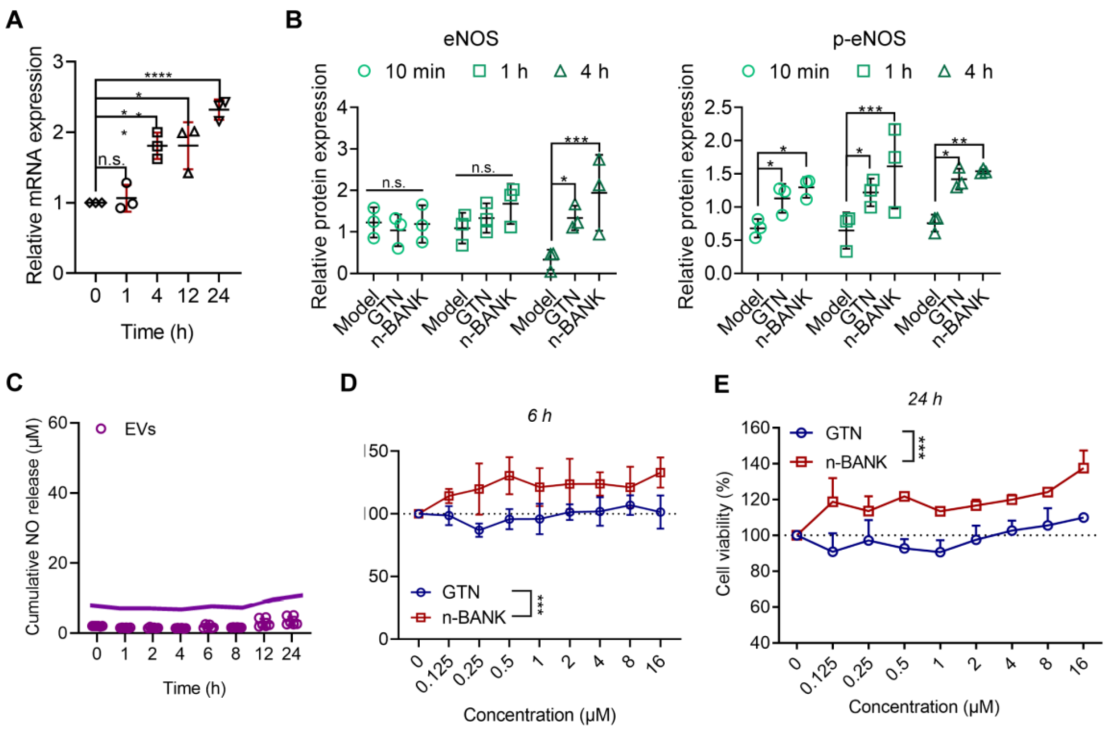

Supplementary Figure 9. Effect of n-BANKs on eNOS phosphorylation and expression. (A) eNOS mRNA levels in SVEC4-10 cells (n = 3). (B) Total and phosphorylated protein levels of eNOS in SVEC4-10 cells (n = 3). (C) Release of NO in the cell supernatants of SVEC4-10 cells treated with EVs was quantified using the Griess reagent (n = 6). (D) SDH activities and (E) cell viabilities of SVEC4-10 cells treated with GTN or n-BANKs (n = 6). SVEC4-10 cells were exposed to hypoxia for 12 hours prior to treatment. Data are mean ± SD, * is $P < 0.05$, ** is $P < 0.01$, *** is $P < 0.001$, **** is $P < 0.0001$, n.s. is $P > 0.05$ by one-way or two-way ANOVA test.

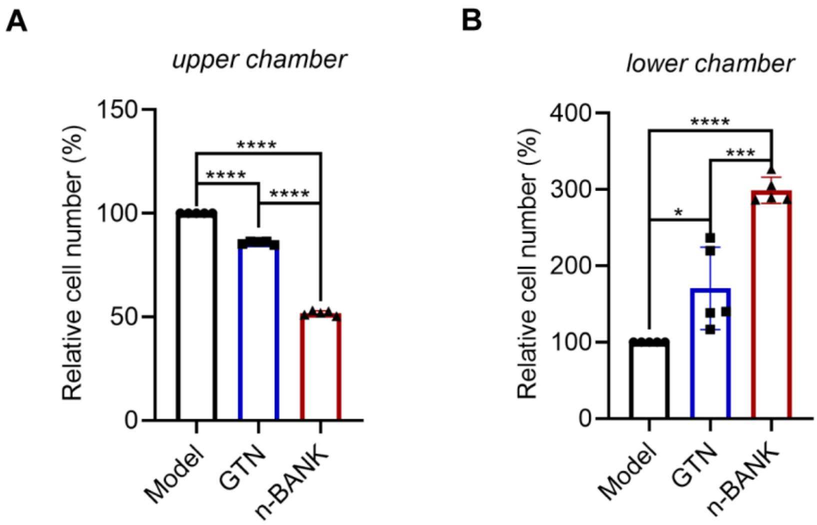

Supplementary Figure 10. Quantification of cells in (A) the upper chambers and (B) the lower champers (n = 5). Data are mean ± SD, * is P < 0.05, *** is P < 0.001, **** is P < 0.0001 by one-way ANOVA test.

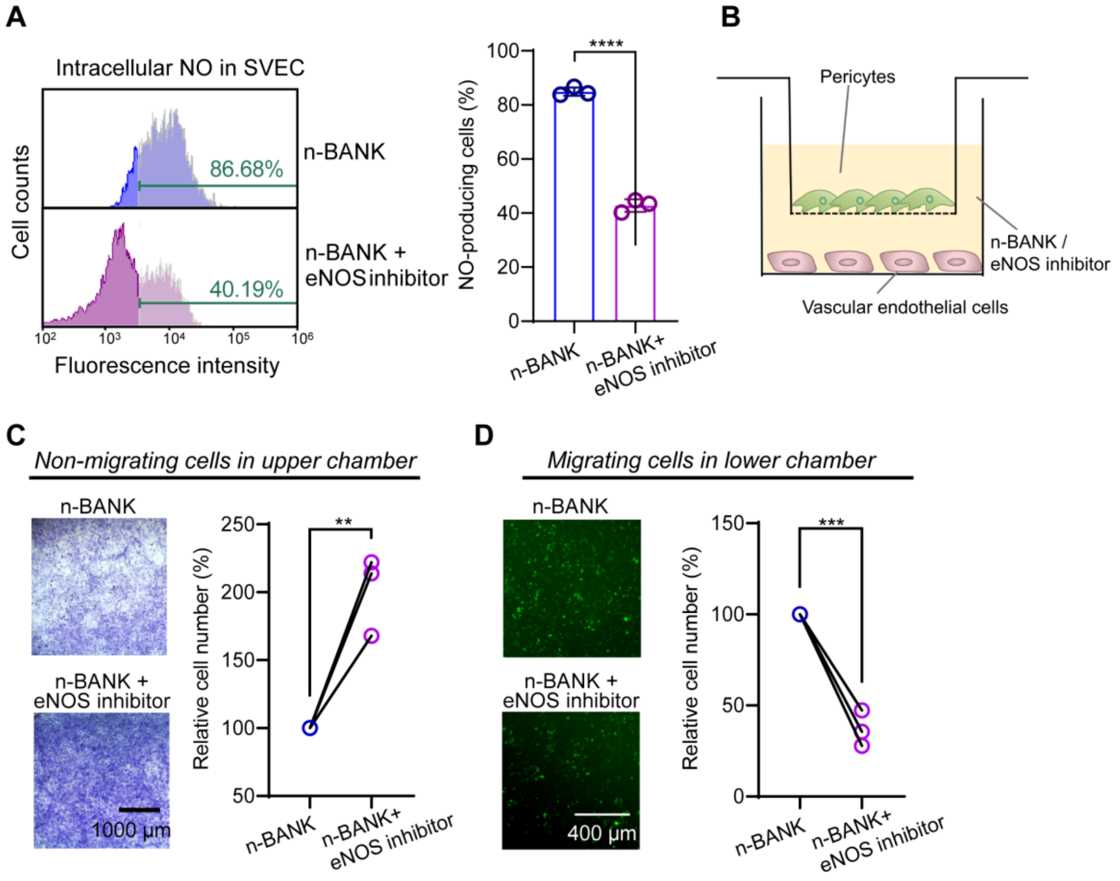

Supplementary Figure 11. eNOS-NO signaling axis functions induced by n-BANKs for pericyte-endothelial interactions. (A) Flow cytometry analysis of NO levels in SVEC4-10 cells (n = 3). (B) Schematic illustration of cell migration assays by an 8.0 μm transwell. (C) The non-migrating cells in the upper chambers were stained by crystal violet and (D) the migrating cells in the lower chambers were stained with calcein acetoxymethyl ester (calcein AM) (n = 3). All data are expressed as mean ± SD, ** is $P < 0.01$, *** is $P < 0.001$, **** is $P < 0.0001$ by Student's t-test.

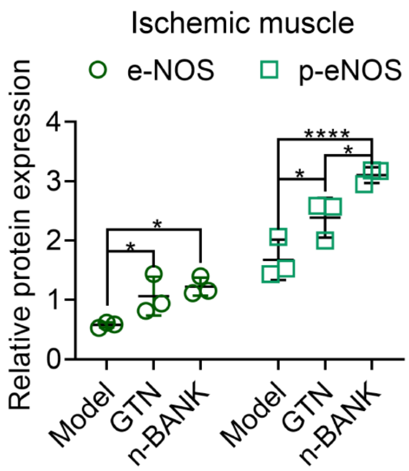

Supplementary Figure 12. Quantification of total and phosphorylated protein levels of eNOS in muscle from ischemic hindlimb (n = 3). Data are mean ± SD, * is $P < 0.05$, **** is $P < 0.0001$ by two-way ANOVA test.

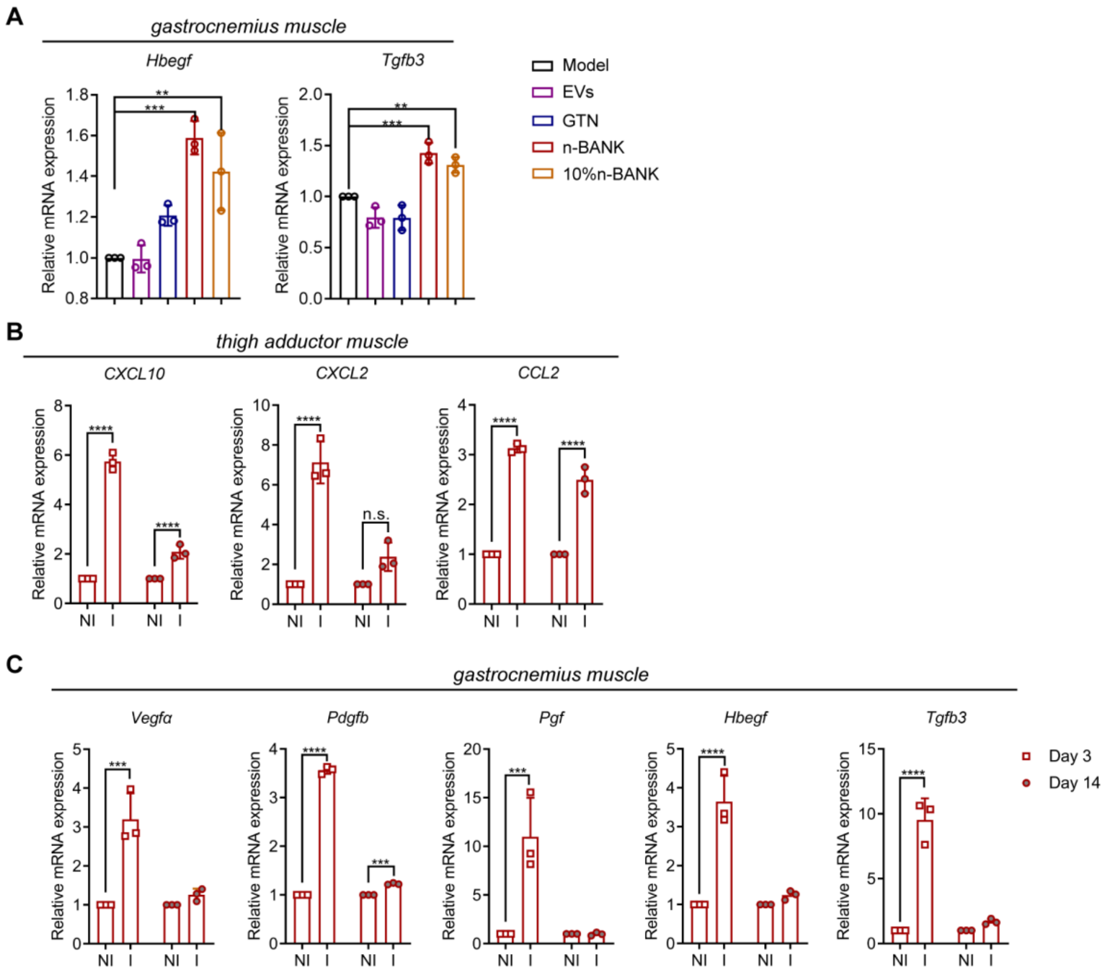

Supplementary Figure 13. qRT-PCR analysis of (A) growth factor genes (Hbegf and Tgfb3) on gastrocnemius muscle, (B) chemokine genes (CXCL10, CXCL2 and CCL2) on thigh adductor muscle and (C) growth factor genes (Vegfα, Pdgfb, Pgf, Hbegf and Tgfb3) on gastrocnemius muscle (n = 3). Data are mean ± SD, ** is $P < 0.01$, *** is $P < 0.001$, **** is $P < 0.0001$ by one-way or two-way ANOVA test.

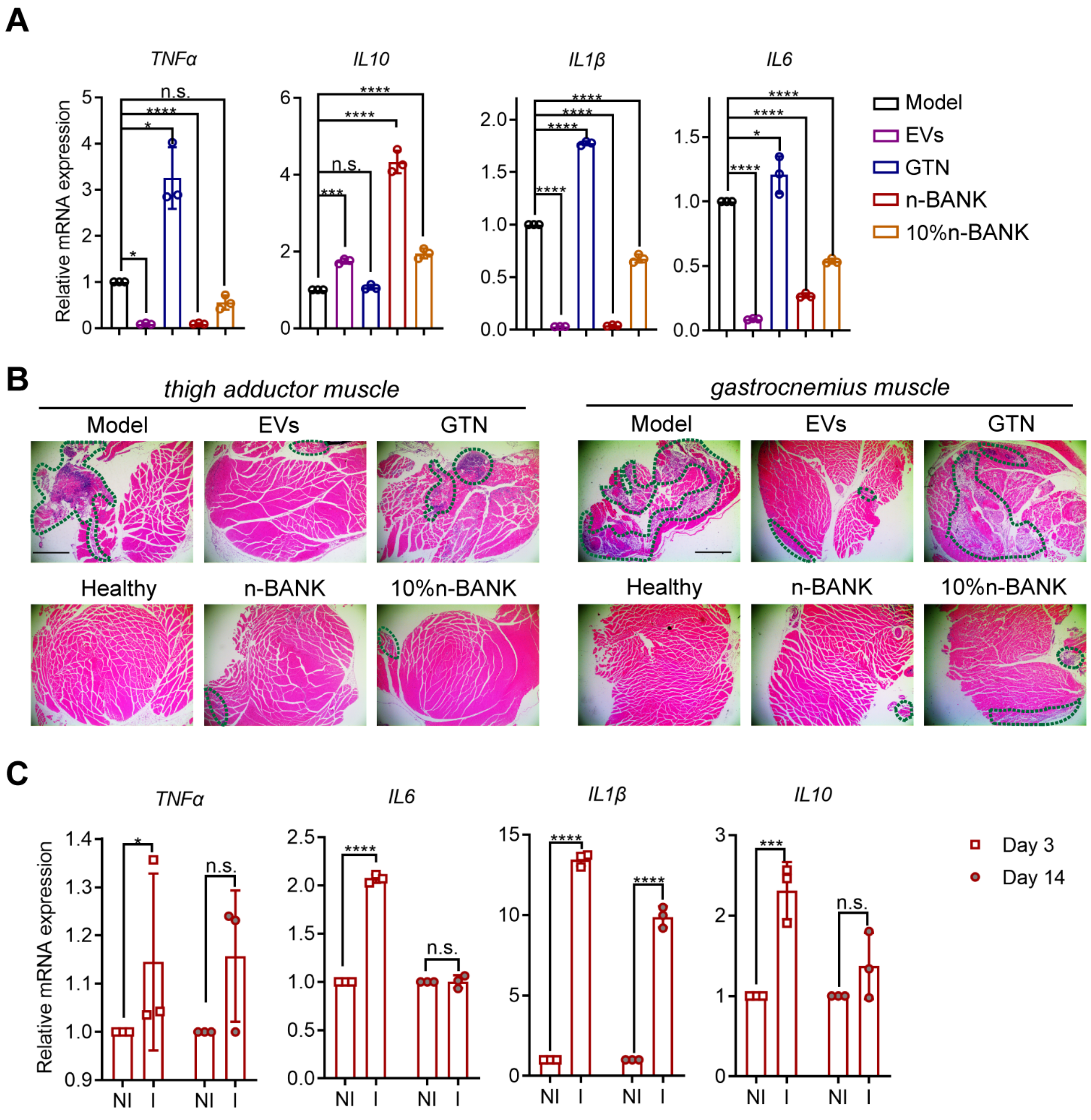

Supplementary Figure 14. n-BANKs prevented ischemia-induced proinflammatory cytokine cascade. (A) qRT–PCR analysis of muscle tissues harvested from ischemic hindlimbs for the genes encoding the inflammatory cytokines (TNFα, IL10, IL1β and IL6) at day 3 (n = 3). (B) H&E staining of histological ischemic hindlimb sections at day 3 after surgery. Scale bar, 1000 μm. (C) Expression of inflammatory cytokines (TNFα, IL10, IL1β and IL6) in muscle from non-ischemic (NI) and ischemic (I) hindlimbs of n-BANK treated mice at day 3 and day 14 (n = 3). Data are mean ± SD, *

is $P < 0.05$, *** is $P < 0.001$, **** is $P < 0.0001$, n.s. is $P > 0.05$ by one-way or two-way ANOVA test.

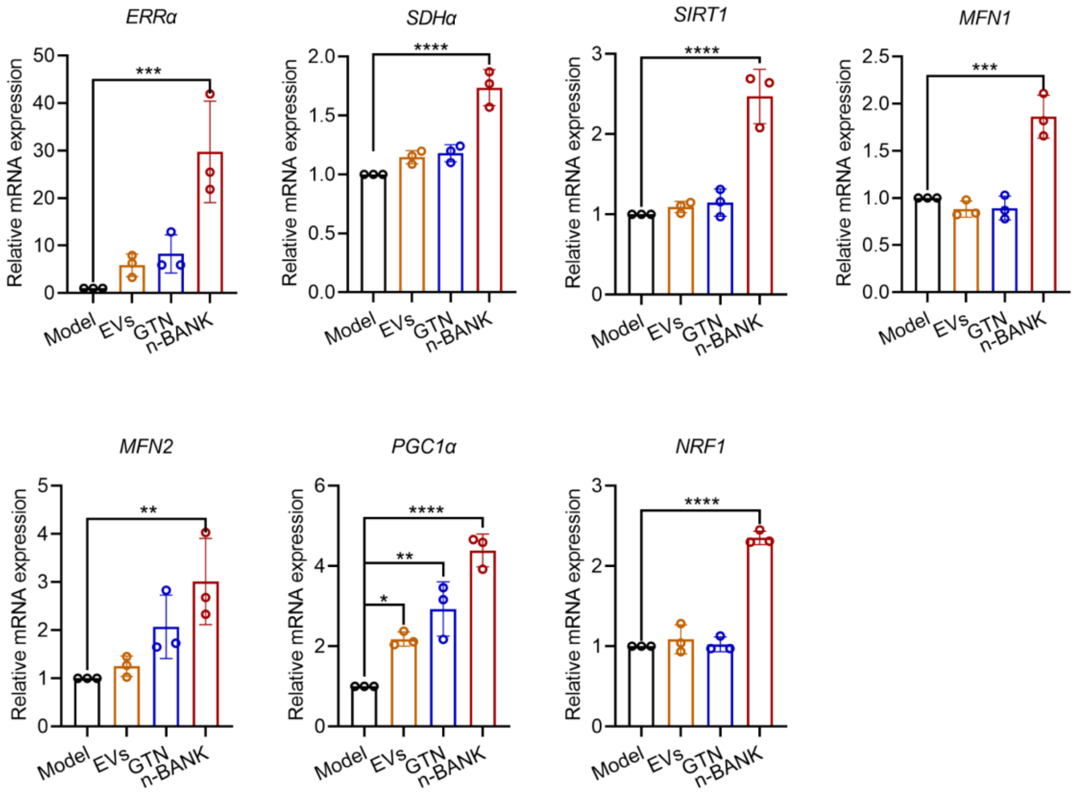

Supplementary Figure 15. qRT-PCR analysis of mitochondrial biogenesis-related genes in muscle from ischemic hindlimbs at day 3 after treatment (n = 3). Data are mean ± SD, * is $P < 0.05$, ** is $P < 0.01$, *** is $P < 0.001$, **** is $P < 0.0001$ by one-way ANOVA test.

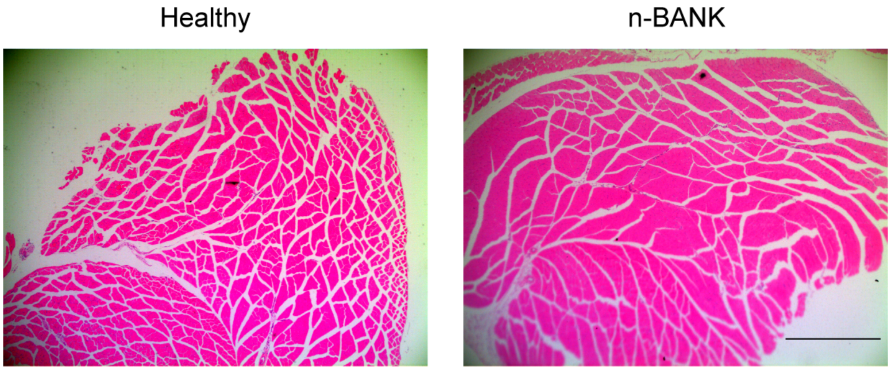

Supplementary Figure 16. Representative H&E staining of histological ischemic hindlimb sections at day 14 after surgery. Scale bar, 1000 μm.

Supplementary Table 1. Primers (FW, Forward; REV, Reverse) used in this study

Gene	Sequence
Fibroblast growth factor 2 (FGF2)-FW	GAGCGACCCACACGTCAAACTAC
Fibroblast growth factor 2 (FGF2)-REV	CAGCCGTCCATCTTCCTTCATAGC
Insulin like growth factor 1 (IGF1)-FW	GCTCTGCTTGCTCACCTTCACC
Insulin like growth factor 1 (IGF1)-REV	CGGTCCACACACGAACTGAAGAG
Transforming growth factor beta (TGF-β)-FW	ACCGCAACAACGCCATCTATGAG
Transforming growth factor beta (TGF-β)-REV	GGCACTGCTTCCCGAATGTCTG
Vascular endothelial growth factor (VEGF)-FW	TGACGGACAGACAGACAGACACC
Vascular endothelial growth factor (VEGF)-REV	ACGGCTACTACGGAGCGAGAAG
Hepatocyte growth factor (HGF)-FW	GGATTCGCAGTACCCTCACAAGC
Hepatocyte growth factor (HGF)-REV	AGCAGTAGCCAACTCGGATGTTTG
Nerve growth factor (NGF)-FW	GACCACAGCCACAGACATCAAGG
Nerve growth factor (NGF)-REV	GGCACCCACTCTCAACAGGATTG
C-X-C motif chemokine ligand 10 (CXCL10)-FW	CAACTGCATCCATATCGATGAC
C-X-C motif chemokine ligand 10 (CXCL10)-REV	GATTCCGGATTCAGACATCTCT
C-X-C motif chemokine ligand 2 (CXCL2)-FW	GGTTGACTTCAAGAACATCCAG
C-X-C motif chemokine ligand 2 (CXCL2)-REV	TTGAGAGTGGCTATGACTTCTG
C-C motif chemokine ligand 2 (CCL2)-FW	AGAATCACCAGCAGCAAGTGTCC
C-C motif chemokine ligand 2 (CCL2)-REV	TTGCTTGTCCAGGTGGTCCATG
Endothelia nitric oxide synthase (eNOS)-FW	CTGAGAGCCTGCAATTACTACC
Endothelia nitric oxide synthase (eNOS)-REV	TTTCCACAGAGAGGATTGTAGC
CD31-FW	CACAACAAACAAGCTAGCAAGA
CD31-REV	TTTGGCTGCAACTATTAAGGTG
CD34-FW	GTTATTTCCTGATGAACCGTCG
CD34-REV	CTCCACCATTCTCCGTGTAATA

CD105-FW	TCATGACTCTGGCACTCAATAA
CD105-REV	CACTGTACCTTTTTCCGAAGTG
Tumor necrosis factor α (TNFα)-FW	GCGACGTGGAACTGGCAGAAG
Tumor necrosis factor α (TNFα)-REV	GAATGAGAAGAGGCTGAGACATAGGC
Interleukin 1 beta (IL1β)-FW	TCGCAGCAGCACATCAACAAGAG
Interleukin 1 beta (IL1β)-REV	TGCTCATGTCCTCATCCTGGAAGG
Interleukin 6 (IL6)-FW	CTCCCAACAGACCTGTCTATAC
Interleukin 6 (IL6)-REV	CCATTGCACAACTCTTTTCTCA
Interleukin 10 (IL10)-FW	TTCTTTCAAACAAAGGACCAGC
Interleukin 10 (IL10)-REV	GCAACCCAAGTAACCCTTAAAG
Heparin binding EGF like growth factor (Hbegf)-FW	GTTGCCCAAGTGAATTACTCTG
Heparin binding EGF like growth factor (Hbegf)-REV	TCGTCTGGATGGTCATTTCAC
Placental growth factor (Pgf)-FW	CCATCTTTCAGGTCCCTAGATC
Placental growth factor (Pgf)-REV	GCTCCGTACATACTTAAGGTGA
Platelet derived growth factor subunit B (Pdgfb)-FW	GCATTCTTGTGGTCGGAGGAGTG
Platelet derived growth factor subunit B (Pdgfb)-REV	TGGTCCAGGTCAGTCGCTCATAG
Estrogen related receptor alpha (ERRα)-FW	GGCACAAGGAGGAGGAGGATGG
Estrogen related receptor alpha (ERRα)-REV	AGGCAGAGGCGTTTGGGTAGAG
Succinate dehydrogenase α subunit (SDHα)-FW	ACATCAGAACTACGCCTAAACA
Succinate dehydrogenase α subunit (SDHα)-REV	TTTCACAGCCTTCTTGCAATAC
Sirtuin 1 (SIRT1)-FW	CGCTGTGGCAGATTGTTATTAA
Sirtuin 1 (SIRT1)-REV	TTGATCTGAAGTCAGGAATCCC
Mitofusin 1 (MFN1)-FW	CCATCTTTCAGGTCCCTAGATC
Mitofusin 1 (MFN1)-REV	GCTCCGTACATACTTAAGGTGA
Mitofusin 2 (MFN2)-FW	GCATTCTTGTGGTCGGAGGAGTG

Mitofusin 2 (MFN2)-REV	TGGTCCAGGTCAGTCGCTCATAG
Peroxisome proliferator-activated receptor gamma, coactivator 1 alpha (PGC1α)-FW	GGATATACTTTACGCAGGTCGA
Peroxisome proliferator-activated receptor gamma, coactivator 1 alpha (PGC1α)-REV	CGTCTGAGTTGGTATCTAGGTC
Nuclear respiratory factor 1 (NRF1)-FW	GTTGCCCAAGTGAATTACTCTG
Nuclear respiratory factor 1 (NRF1)-REV	TCGTCTGGATGGTCATTTCAC
Glyceraldehyde-3-phosphate dehydrogenase (GAPDH)-FW	GATCATCAGCAATGCCTCCT
Glyceraldehyde-3-phosphate dehydrogenase (GAPDH)-REV	TGTGGTCATGAGTCCTCCCA
