# Supplementary material for: Enhanced pericyte-endothelial interactions through NO-boosted extracellular vesicles drive revascularization in a mouse model of ischemic injury
Source: Nat Commun. 2023 Nov 13;14:7334. doi: 10.1038/s41467-023-43153-x (PMC10643472; doi:10.1038/s41467-023-43153-x)
Supplement: Supplementary file 3 — Description of Additional Supplementary Files Document [file 41467_2023_43153_MOESM3_ESM.pdf]

Description of Additional Supplementary Files

Supplementary Movie 1 to 9

Supplementary Movie 1, videos of blood flow in mice treated with PBS.

Supplementary Movie 2, videos of blood flow in mice treated with EVs.

Supplementary Movie 3, videos of blood flow in mice treated with GTN.

Supplementary Movie 4, videos of blood flow in mice treated with n-BANK.

Supplementary Movie 5, videos of blood flow in mice treated with 10% n-BANK.

Supplementary Movie 6, videos for hind limb function detection of mice in the n-BANK group at day 14 after treatment.

Supplementary Movie 7, videos for hind limb function detection of mice in the healthy group at day 14 after treatment.

Supplementary Movie 8, videos for hind limb function detection of mice in the 10% n-BANK group at day 14 after treatment.

Supplementary Movie 9, videos for hind limb function detection of mice in the GTN group at day 14 after treatment.
