# Supplementary material for: Enhanced pericyte-endothelial interactions through NO-boosted extracellular vesicles drive revascularization in a mouse model of ischemic injury
Source: Nat Commun. 2023 Nov 13;14:7334. doi: 10.1038/s41467-023-43153-x (PMC10643472; doi:10.1038/s41467-023-43153-x)
Supplement: Supplementary file 13 — Reporting Summary [file 41467_2023_43153_MOESM13_ESM.pdf]

nature portfolio

Corresponding author(s): Min Feng

Last updated by author(s): Oct 22, 2023

Reporting Summary

Nature Portfolio wishes to improve the reproducibility of the work that we publish. This form provides structure for consistency and transparency in reporting. For further information on Nature Portfolio policies, see our Editorial Policies and the Editorial Policy Checklist.

Statistics

For all statistical analyses, confirm that the following items are present in the figure legend, table legend, main text, or Methods section.

n/a	Confirmed	
☐	☒	The exact sample size (*n*) for each experimental group/condition, given as a discrete number and unit of measurement
☐	☒	A statement on whether measurements were taken from distinct samples or whether the same sample was measured repeatedly
☐	☒	The statistical test(s) used AND whether they are one- or two-sided<br>*Only common tests should be described solely by name; describe more complex techniques in the Methods section.*
☒	☐	A description of all covariates tested
☐	☒	A description of any assumptions or corrections, such as tests of normality and adjustment for multiple comparisons
☐	☒	A full description of the statistical parameters including central tendency (e.g. means) or other basic estimates (e.g. regression coefficient) AND variation (e.g. standard deviation) or associated estimates of uncertainty (e.g. confidence intervals)
☐	☒	For null hypothesis testing, the test statistic (e.g. *F*, *t*, *r*) with confidence intervals, effect sizes, degrees of freedom and *P* value noted<br>*Give P values as exact values whenever suitable.*
☒	☐	For Bayesian analysis, information on the choice of priors and Markov chain Monte Carlo settings
☒	☐	For hierarchical and complex designs, identification of the appropriate level for tests and full reporting of outcomes
☒	☐	Estimates of effect sizes (e.g. Cohen's *d*, Pearson's *r*), indicating how they were calculated

Our web collection on statistics for biologists contains articles on many of the points above.

Software and code

Policy information about availability of computer code

Data collection	The flow cytometry data were acquired by CytoFLEX-S (Beckman Coulter, USA). Particle size and zeta potential were performed on Nanosight NS300 system (Malvern, UK) and Zetasizer Nano-2S (Malvern, UK). Transmission electron microscopy images were acquired by JEM-1400 electron microscope (JEOL, Tokyo, Japan). Surface plasmon resonance analysis was conducted by Biacore 8K (GE healthcare, Sweden). Fluorescence spectra were recorded on a fluorescence spectrometer Fluoromax-4 (HORIBA, USA). The fluorescence imaging of n-BANKs was performed on a scanning confocal microscope (AXR, Nikon, Japan). Quantitative real-time PCR was performed on a Bio-Rad MyiQ Real-Time PCR Detection System (Bio-Rad Laboratories). EVOS FL Auto Cell Imaging System (Life Technologies) was used in cell tube formation assay, in vitro wound healing assay and histological analysis. Pericyte migration was monitored in a CIM-16 well plate by using xCELLigence real time cell analyzer (RTCA) instrument (ACEA Biosciences, San Diego, CA, USA). Immunofluorescence images of the tissues were captured on an Olympus Fluoview confocal microscope (FV3000, Olympus, Japan). Limb perfusion was monitored with a laser Doppler perfusion imaging system (Perimed, Inc., Ardmore, PA). Serum biochemical analysis was performed using an automatic biochemical analyzer (HITACHI, Japan).
Data analysis	All statistical analysis was performed using Prism 7.0 software (GraphPad Software). Flow cytometry data was analyzed by CytExpert 2.3.1 (Beckman Coulter, USA). Molecular docking were generated by DiscoverStudio(Version 2020) and PyMOL (Version 1.7).

For manuscripts utilizing custom algorithms or software that are central to the research but not yet described in published literature, software must be made available to editors and reviewers. We strongly encourage code deposition in a community repository (e.g. GitHub). See the Nature Portfolio guidelines for submitting code & software for further information.

Data

Policy information about availability of data

All manuscripts must include a data availability statement. This statement should provide the following information, where applicable:

- Accession codes, unique identifiers, or web links for publicly available datasets
- A description of any restrictions on data availability
- For clinical datasets or third party data, please ensure that the statement adheres to our policy

The data supporting the findings from this study are available within the Article, Supplementary Information, or Source Data file. Source data are provided with this paper.

Research involving human participants, their data, or biological material

Policy information about studies with human participants or human data. See also policy information about sex, gender (identity/presentation), and sexual orientation and race, ethnicity and racism.

Reporting on sex and gender	Not human research.
Reporting on race, ethnicity, or other socially relevant groupings	Not human research.
Population characteristics	Not human research.
Recruitment	Not human research.
Ethics oversight	Not human research.

Note that full information on the approval of the study protocol must also be provided in the manuscript.

Field-specific reporting

Please select the one below that is the best fit for your research. If you are not sure, read the appropriate sections before making your selection.

☒ Life sciences ☐ Behavioural & social sciences ☐ Ecological, evolutionary & environmental sciences

For a reference copy of the document with all sections, see nature.com/documents/nr-reporting-summary-flat.pdf

Life sciences study design

All studies must disclose on these points even when the disclosure is negative.

Sample size	Sample sizes in this study were based on previous studies with similar experiments, e.g. PMID: 31530804 (Kant et al., 2019, Nat Commun.) ; PMID: 32001693 (Lee et al., 2020, Nat Commun.); PMID: 33536281 (Wang et al., 2021, Sci Transl Med.). Sample sizes for each experiment were described in figure legends. For all experiments in this study, n ≥ 3.
Data exclusions	No data were excluded form the manuscript.
Replication	Experiments were repeated with the same conditions. The number of repeats were indicated in the figure legends.
Randomization	The samples and animals used in this study were distributed in to several groups randomly and equally.
Blinding	All the investigators were blinded during data collection and analysis for in vitro and in vivo experiments.

Reporting for specific materials, systems and methods

We require information from authors about some types of materials, experimental systems and methods used in many studies. Here, indicate whether each material, system or method listed is relevant to your study. If you are not sure if a list item applies to your research, read the appropriate section before selecting a response.

Materials & experimental systems

n/a	Involved in the study
	☒ Antibodies
	☒ Eukaryotic cell lines
☒	Palaeontology and archaeology
	☒ Animals and other organisms
☒	Clinical data
☒	Dual use research of concern
☒	Plants

Methods

n/a	Involved in the study
☒	ChIP-seq
	☒ Flow cytometry
☒	MRI-based neuroimaging

Antibodies

Antibodies used

1. APC-R700 rat anti-mouse CD45 antibody, Clone: 30-F11 (BD Biosciences, Cat# 565478, dilution 1:80 for flow cytometry);
2. APC anti-mouse/human CD11b antibody, Clone: M1/70 (BioLegend, Cat# 101211, dilution 1:80 for flow cytometry);
3. FITC anti-mouse/rat CD29 antibody, Clone: HMβ1-1 (BioLegend, Cat# 102205, dilution 1:50 for flow cytometry);
4. FITC anti-mouse Ly-6A/E (Sca-1) antibody, Clone: E13-161.7 (BioLegend, Cat# 122505, dilution 1:50 for flow cytometry);
5. Recombinant Anti-VEGFα antibody [EPR20705] (Abcam, Cat# ab214424, dilution 1:1000 for western blot);
6. Recombinant Anti-Von Willebrand Factor antibody [EPR12011] (Abcam, Cat# ab174290, dilution 1:1000 for western blot);
7. Recombinant Anti-eNOS antibody [EPR19296] (Abcam, Cat# ab199956, dilution 1:1000 for western blot);
8. Recombinant Anti-eNOS (phospho S1177) antibody [EPR20991] (Abcam, Cat# ab215717, dilution 1:1000 for western blot);
9. Anti-beta Actin antibody (Abcam, Cat# ab8227, dilution 1:1000 for western blot);
10. Anti-beta Tubulin antibody - Loading Control (Abcam, Cat# ab6046, dilution 1:500 for western blot);
11. Anti-GAPDH antibody - Loading Control (Abcam, Cat# ab9485, dilution 1:2500 for western blot);
12. Goat Anti-Rabbit IgG H&L (HRP) (Abcam, Cat# ab6721, dilution 1:2000 for western blot);
13. Anti-CD31 Rabbit pAb (Servicebio, Cat# GB11063-2-100, dilution 1:200 for immunofluorescence);
14. Anti-NG2 Rabbit pAb (Servicebio, Cat# GB115534-100, dilution 1:200 for immunofluorescence);
15. Anti-alpha smooth muscle Actin (α-SMA) Rabbit pAb (Servicebio, Cat# GB111364-100, dilution 1:300 for immunofluorescence);
16. Anti-eNOS Mouse mAb (Servicebio, Cat# GB12086-100, dilution 1:500 for immunofluorescence);
17. FITC conjugated Goat Anti-Rabbit IgG (H+L) (Servicebio, Cat# GB22303, dilution 1:100 for immunofluorescence);
18. Cy3 conjugated Goat Anti-Rabbit IgG (H+L) (Servicebio, Cat# GB21303, dilution 1:200 for immunofluorescence);
19. FITC conjugated Goat Anti-Mouse IgG (H+L) (Servicebio, Cat# GB22301, dilution 1:100 for immunofluorescence).

Validation

All the antibodies used in this study are commercially available. The information of these antibodies are available in the manufacture's website.
1. APC-R700 rat anti-mouse CD45 antibody: https://www.bdbiosciences.com/en-us/products/reagents/flow-cytometry-reagents/research-reagents/single-color-antibodies-ruo/apc-r700-rat-anti-mouse-cd45.565478;
2. APC anti-mouse/human CD11b antibody: https://www.biolegend.com/en-us/products/apc-anti-mouse-human-cd11b-antibody-345;
3. FITC anti-mouse/rat CD29 antibody: https://www.biolegend.com/en-us/products/fitc-anti-mouse-rat-cd29-antibody-2258;
4. FITC anti-mouse Ly-6A/E (Sca-1) antibody: https://www.biolegend.com/en-us/products/fitc-anti-mouse-ly-6a-e-sca-1-antibody-3894;
5. Recombinant Anti-VEGFα antibody: https://www.abcam.com/products/primary-antibodies/vegfa-antibody-epr20705-ab214424.html;
6. Recombinant Anti-Von Willebrand Factor antibody: https://www.abcam.com/products/primary-antibodies/von-willebrand-factor-antibody-epr12011-ab174290.html;
7. Recombinant Anti-eNOS antibody: https://www.abcam.com/products/primary-antibodies/enos-antibody-epr19296-ab199956.html;
8. Recombinant Anti-eNOS (phospho S1177) antibody: https://www.abcam.com/products/primary-antibodies/enos-phospho-s1177-antibody-epr20991-ab215717.html;
9. Anti-beta Actin antibody: https://www.abcam.com/products/primary-antibodies/beta-actin-antibody-ab8227.html;
10. Anti-beta Tubulin antibody - Loading Control: https://www.abcam.com/products/primary-antibodies/beta-tubulin-antibody-loading-control-ab6046.html;
11. Anti-GAPDH antibody - Loading Control: https://www.abcam.com/products/primary-antibodies/gapdh-antibody-loading-control-ab9485.html;
12. Goat Anti-Rabbit IgG H&L (HRP): https://www.abcam.com/products/secondary-antibodies/goat-rabbit-igg-hl-hrp-ab6721.html;
13. Anti-CD31 Rabbit pAb: https://www.servicebio.cn/goodsdetail?id=1345;
14. Anti-NG2 Rabbit pAb: https://www.servicebio.cn/goodsdetail?id=20492;
15. Anti-alpha smooth muscle Actin (α-SMA) Rabbit pAb: https://www.servicebio.cn/goodsdetail?id=3743;
16. Anti-eNOS Mouse mAb: https://www.servicebio.cn/goodsdetail?id=2651;
17. FITC conjugated Goat Anti-Rabbit IgG (H+L): https://www.servicebio.cn/goodsdetail?id=259;
18. Cy3 conjugated Goat Anti-Rabbit IgG (H+L): https://www.servicebio.cn/goodsdetail?id=253;
19. FITC conjugated Goat Anti-Mouse IgG (H+L): https://www.servicebio.cn/goodsdetail?id=257.

Eukaryotic cell lines

Policy information about cell lines and Sex and Gender in Research

Cell line source(s)

SVEC4-10 cell line (CRL-2181) was purchased from the American Tissue Culture Collection (ATCC).

Authentication	The cell line was authenticated by short tandem repeat (STR) profiling.
Mycoplasma contamination	We tested and confirmed that the cell line was mycoplasma negative.
Commonly misidentified lines (See ICLAC register)	NO commonly misidentified lines were used in this study.

Animals and other research organisms

Policy information about studies involving animals; ARRIVE guidelines recommended for reporting animal research, and Sex and Gender in Research

Laboratory animals	Female BALB/c mice at 8-10 weeks of age were purchased from the Laboratory Animal Center, Sun Yat-sen University. Efforts were made to minimize animal suffering, and the necessary sample size was calculated before the experiments to reduce the number of animals used per condition. All the animals were housed under specific pathogen free (SPF) barrier environment (20-26°C and at 40%–70% humidity), under the 12h/12h dark/light cycle. All mice had free access to standardized food and water. Isoflurane (2%, 0.5 L/min) were used in animal euthanasia practice.
Wild animals	Our study did not involve wild animals.
Reporting on sex	Female BALB/c mice were used in this study. Data from the Global Burden of Disease Study suggests that peripheral arterial disease cases in women outnumber those in men across all age groups (Roth et al., 2020, J Am Coll Cardiol.) [PMID: 33309175]. Particularly, the prevalence of peripheral arterial disease in younger women in low-income or middle-income countries was 2.97 times more than that of men of the same age (Fowkes et al., 2013, Lancet) [PMID: 23915883]. Critical limb ischemia (CLI) is a severe form of peripheral arterial disease. Thus, female mice were used to construct a CLI mouse model.
Field-collected samples	Our study did not involve field-collected samples.
Ethics oversight	All the animal experiments conducted strictly observed the Guiding Principles for the Use of Laboratory Animals and were approved by the Institutional Animal Care and Use Committee of the Sun Yat-sen University, ethical approval number No.44008500026874.

Note that full information on the approval of the study protocol must also be provided in the manuscript.

Flow Cytometry

Plots

Confirm that:

☒ The axis labels state the marker and fluorochrome used (e.g. CD4-FITC).

☒ The axis scales are clearly visible. Include numbers along axes only for bottom left plot of group (a 'group' is an analysis of identical markers).

☒ All plots are contour plots with outliers or pseudocolor plots.

☒ A numerical value for number of cells or percentage (with statistics) is provided.

Methodology

Sample preparation	For MSC identification, cells were incubated with APC-R700 anti-CD45 antibody, APC anti-CD11b antibody, FITC anti-CD29 antibody and FITC anti-Sca-1 antibody for 30 min on ice, respectively. And then the cells were washed and collected for flow cytometry. Mitochondrial membrane potential (MMP) was detected by JC-1 staining assay. After treatment, SVEC4-10 cells were stained with 2 µM JC-1 for 30 min at 37 °C. Then the cells were washed and collected for flow cytometry. For detection of intracellular NO generation, SVEC4-10 cells were first treated with drugs and then incubated with DAF-FM DA solution (dilution 1:1000) for 20 min at 37 °C. Cells were then washed and collected by centrifugation.
Instrument	Flow cytometry (CytoFLEX-S, Beckman Counter)
Software	CytExpert 2.3.1 (Beckman Coulter, USA)
Cell population abundance	FACS analysis was performed on each sample to a total number for at least 10,000 events.
Gating strategy	Cells were first gated by FSC/SSC to identify the cell population of interest. The cells were then gated using appropriate channels by gating unstained cells and single stained cells.

☒ Tick this box to confirm that a figure exemplifying the gating strategy is provided in the Supplementary Information.
